# Identification of clinical trait–related lncRNA and mRNA biomarkers with weighted gene co-expression network analysis as useful tool for personalized medicine in ovarian cancer

**DOI:** 10.1007/s13167-019-00175-0

**Published:** 2019-07-19

**Authors:** Na Li, Xianquan Zhan

**Affiliations:** 1 Key Laboratory of Cancer Proteomics of Chinese Ministry of Health, Xiangya Hospital, Central South University, 87 Xiangya Road, Changsha, Hunan 410008 People’s Republic of China; 2 Hunan Engineering Laboratory for Structural Biology and Drug Design, Xiangya Hospital, Central South University, 87 Xiangya Road, Changsha, Hunan 410008 People’s Republic of China; 3 State Local Joint Engineering Laboratory for Anticancer Drugs, Xiangya Hospital, Central South University, 87 Xiangya Road, Changsha, Hunan 410008 People’s Republic of China; 4 National Clinical Research Center for Geriatric Disorders, Xiangya Hospital, Central South University, 88 Xiangya Road, Changsha, Hunan 410008 People’s Republic of China

**Keywords:** Ovarian cancer, Co-expression module, lncRNA, mRNA, Ovarian cancer, WGCNA, Diagnosis, Prognostic assessment, Predictive preventive personalized medicine (PPPM)

## Abstract

**Relevance:**

The pathogenesis and biomarkers of ovarian cancer (OC) remain not well-known in diagnosis, effective therapy, and prognostic assessment in OC personalized medicine. The novel identified lncRNA and mRNA biomarkers from gene co-expression modules associated with clinical traits provide new insight for effective treatment of ovarian cancer.

**Purpose:**

Long non-coding RNAs (lncRNAs) are relevant to tumorigenesis via multiple mechanisms. This study aimed to investigate cancer-specific lncRNAs and mRNAs, and their related networks in OCs.

**Methods:**

This study comprehensively analyzed lncRNAs and mRNAs with associated competing endogenous RNA (ceRNA) network and lncRNA–RNA binding protein–mRNA network in the OC tissues in the Cancer Genome Atlas, including 2562 cancer-specific lncRNAs (*n* = 352 OC tissues) and 5000 mRNAs (*n* = 359 OC tissues). The weighted gene co-expression network analysis (WGCNA) was used to construct the co-expression gene modules and their relationship with clinical traits. The statistically significant difference of identified lncRNAs and mRNAs was confirmed with qRT-PCR in OC cells.

**Results:**

An lncRNA-based co-expression module was significantly correlated with patient age at initial pathologic diagnosis, lymphatic invasion, tissues source site, and vascular invasion, and identified 16 lncRNAs (ACTA2-AS1, CARD8-AS1, HCP5, HHIP-AS1, HOTAIRM1, ITGB2-AS1, LINC00324, LINC00605, LINC01503, LINC01547, MIR31HG, MIR155HG, OTUD6B-AS1, PSMG3-AS1, SH3PXD2A-AS1, and ZBED5-AS1) that were significantly related to overall survival in OC patients. An mRNA-based co-expression module was significantly correlated with patient age at initial pathologic diagnosis, lymphatic invasion, tumor residual disease, and vascular invasion; and identified 21 hub-mRNA molecules and 11 mRNAs (FBN3, TCF7L1, SBK1, TRO, TUBB2B, PLCG1, KIAA1549, PHC1, DNMT3A, LAMA1, and C10orf82) that were closely linked with OC patients’ overall survival. Moreover, the prognostic model of five-gene signature (OTUD6B-AS1, PSMG3-AS1, ZBED5-AS1, SBK1, and PLCG1) was constructed to predict risk score in OC patients. Furthermore, starBase bioinformatics constructed the lncRNA–miRNA–mRNA and lncRNA–RNA binding protein-mRNA networks in OCs.

**Conclusion:**

These new findings showed that lncRNA-related networks in OCs are a useful resource for identification of biomarkers in OCs.

**Electronic supplementary material:**

The online version of this article (10.1007/s13167-019-00175-0) contains supplementary material, which is available to authorized users.

## Introduction

Ovarian cancer (OC) is a highly malignant tumor with poor prognosis, which is the most deadly cancer in gynecology [1]. Most OC patients are often detected in late clinical stages because the OC pathogenesis is concealed without effective characteristics. In less than 30% of patients, OCs were found to be located in the ovary but most of which spread to pelvic and abdominal organs. Most of the patients have no obvious symptoms in the early stage, and the common symptoms of the patients in the late stage include emaciation, bellyache, abdominal distension, pelvic lumps, and ascites [2]. Despite the continuous improvement made in the diagnosis and treatment of OC, OC is still a serious threat to women’s lives [3]. Currently, the common OC biomarkers included carbohydrate antigen 125 (CA125), human epididymis protein 4 (HE4), breast cancer 1 (BRCA1), and human chorionic gonadotropin (HCG). The diagnosis of OCs based on those common biomarkers is still unsatisfactory. For example, CA125 was not an ideal biomarker due to low sensitivity and high specificity [4]. Even for two-biomarker (CA125 and HE4) joint detection, the sensitivity is only about 71% [5]. Other novel OC biomarkers such as osteopontin (OPN), mesothelin (SMRP), and vascular endothelial growth factor A121 (VEGFA) are still studied in clinical trials [6]. It is an urgent need to explore novel effective tumor molecular biomarkers for early diagnosis, prognosis monitoring, and therapy improvement [7].

OC is a chronic and complex disease that is involved in a series of molecular alterations in genome, transcriptome, proteome, metabolome, and radiome [8–12]. Multiomics has driven the development of predictive, preventive, and personalized medicine (PPPM) in OCs [13, 14], and PPPM is the effective and affordable strategy for OC care [15]. Of them, transcriptome and proteome are the functional performers of genes [16]. Transcriptome includes non-coding RNAs (lncRNAs and microRNAs) and coding RNAs (mRNAs). The mRNAs are the bridge to link the genome with proteome, and lncRNAs regulate transcription and translation of genes associated with various diseases including cancer. Transcriptome-based pattern biomarkers play important roles in management of OC care [11]. It emphasized important scientific value of transcriptomics for PPPM in OCs.

The length of long non-coding RNAs (lncRNAs) is more than 200 nucleotides without significant protein-coding capacity [17]. lncRNAs showed diverse biological characteristics, which were detected as different expressions in different tissues and different expressions in the same tissues at different growth stages [18]. More and more studies found that the relationship between lncRNAs and cancer is complicated [19]. lncRNAs regulate multiple biological functions associated with tumorigenesis and progression in cancer cells, such as angiogenesis, proliferation, immunity adjustment, epigenetic regulation, invasion, and metastasis of tumor [20]. In addition, lncRNAs are involved in the tumorigenesis through multiple mechanisms, including chromatin modification and structure construction, transcriptional regulation, genome-imprinting regulation, protein post-translational regulation or localization, microRNA regulation, ribonucleoprotein complex formation, and endogenous siRNA production [21]. Moreover, a competing endogenous RNA (ceRNA) hypothesis proposed in 2011 described an intricate post-transcriptional regulatory network, which mainly includes lncRNAs, microRNAs, mRNAs, circRNAs, and other types of RNAs [22]. lncRNAs as ceRNAs might be involved in relevant regulatory mechanisms in OCs. Construction of lncRNA–miRNA–mRNA and lncRNA–RNA binding protein-mRNA networks might provide more clues to OC molecular mechanisms.

Weighted gene co-expression network analysis (WGCNA) was widely applied to identify the relationship between gene-based connections and the disease phenotypes based on microarray data or RNA sequencing in different samples [23]. WGCNA was a comprehensive approach to find modules of strong associated genes, to summarize the identified modules with the eigengene network that was one of a set of right singular vectors of a genes × samples matrix that tabulates (e.g., the mRNA or lncRNA expression of the genes across the samples) or a series of intramodular hub genes, to compute the correlation between modules, to calculate the correlation between gene modules and external sample clinical traits with eigengene network methodology, and to plot the scatterplot of gene significance (GS) vs. module membership (MM) [24]. WGCNA identified gene modules with unsupervised hierarchical clustering method that transforms gene expression matrix into different clusters and provides more credible gene functions [25]. Correlation networks, module–trait relationship, and the scatterplot of GS vs. MM that facilitate to further study the identified key modules and genes are successfully applied to explore various disease biological processes to identify potential biomarkers and therapeutic targets [26]. However, WGCNA is rarely used to study OCs for identification of prognostic biomarkers.

The present study collected the RNA sequencing data of OC tissues in The Cancer Genome Atlas (TCGA) database, and investigated OC-specific lncRNA and mRNA modules associated with OC patients’ clinical characteristics. The WGCNA method was modified with the clinical view so that it could be reasonably utilized for meaningful biological interpretation. The hub genes were extracted from the identified clinical-related co-expression modules, and those hub genes included lncRNAs and mRNAs. Analysis of lncRNA–miRNA–mRNA and lncRNA–RNA binding protein-mRNA networks offered new insights into OC molecular mechanisms. These findings provide the scientific evidence and resource for better understanding of the molecular mechanisms of OCs, and for effective diagnosis, prognostic assessment, and treatment in the OC PPPM context.

## Materials and methods

### TCGA data of OC patients

TCGA database (http://cancergenome.nih.gov/) is created by US National Cancer Institute, which includes 20,000 primary cancer and involves genomic, transcriptomic, proteomic, and methylation data. The TCGA platform is publicly available and is free access for anyone to search, download original data, and for integrated analysis, and TCGA is free of copyright for reuse [27]. Level 3 RNA-seq V2 and clinical data were obtained from 419 OC patients in the TCGA database. If one gene generated multiple missing expression values (expression = 0, and more than 20%), it would be removed. Thus, a total of 352 OC patients met the criteria for lncRNA analysis, and 359 OC patients met the criteria for mRNA analysis. Overall survival analysis of identified genes in OC patients was performed with Kaplan–Meier plotter (http://kmplot.com/private/index.php.p= home). Nine OC prognostic factors of OC data were extracted, including age at initial pathologic diagnosis (patients were aged 26 to 89); Karnofsky performance score (KPS) which represents the activities of daily life (independent, semi-independent, or dependent) after the patients received treatment with a ranking range from KPS 100 (perfect) to 0 (death); lymphatic invasion (yes/no); histologic grade (grade 1, grade2, grade 3, and grade X); cancer status (with tumor/tumor-free); clinical stage (stage I–IV); tissue source site (specimen from different sites of the same patient); tumor residual disease (including no macroscopic disease, 1–10 mm, 11–20 mm, and > 20 mm); and vascular invasion (yes/no; an aggressive tumor had struck a major blood vessel).

### Weighted correlation network analysis of lncRNAs and mRNAs

WGCNA was able to distinguish genes into multiple clusters, and further investigate the relationship between co-expression modules and clinical phenotypes. In this study, weighted gene co-expression modules and module–trait relationship were established with lncRNA and mRNA TCGA expression data and corresponding clinical data through the WGCNA platform of R software (http://www.r-project.org/). This analysis process included (i) downloading of raw data from TCGA, (ii) construction of a gene co-expression network by calculating the connection strength between genes, (iii) identification of modules with hierarchical clustering and dynamic tree cut, (iv) construction of module relationships with eigengene networks, and (v) finding the key drivers in interesting modules by intramodular connectivity and causality testing. In this process, the scale-free topology fit index (SFTFI) (scale-free *R*^2^) ranging from 0 to 1 was used to determine a scale-free topology model. The higher SFTFI value (scale-free *R*^2^) means a better fitting degree. In this study, *β* value was soft-threshold (power). When *β* value (range 1 to 20) was at least 3 for lncRNAs and at least 4 for mRNAs, the corresponding scale-free *R*^2^ value was 0.88 for lncRNAs and 0.91 for mRNAs to obtain a good scale-free topology model. In the cluster dendrogram, genes with highly absolute correlations were clustered into the same co-expression module to generate a cluster dendrogram with FlashClust analysis. Then, the cluster dendrogram was transformed into a topology matrix to form the network heatmap plot. Within each module with the number of genes being more than 30, the adjacency matrix algorithm was used to generate the topological overlap matrix (TOM). Heatmap plot was constructed with Heatmap tool to analyze network-interaction strength. The relationships between modules and nine OC prognostic factors (age at initial pathologic diagnosis, Karnofsky performance score, lymphatic invasion, histologic grade, cancer status, clinical stage, tissue source site, tumor residual disease, and vascular invasion) were analyzed with Pearson correlation coefficient (*r*) and visualized by heat map with *p* value < 0.05. Moreover, GS was the mediated *p* value of each gene (GS = lg^P^) in the linear regression between gene expressions and clinical traits. KEEG pathway (https://david.ncifcrf.gov/home.jsp) and Gene Ontology (GO) (http://www.cytoscape.org/) enrichment analyses within mRNA modules were performed to identify OC-related module with *p* value < 0.05. The maximum intramodular connectivity of mRNAs was referred as intramodular hub genes. Furthermore, the OC survival–related lncRNAs in OC-related module (yellow) were plotted expression correlation network with hub genes in OC-related module with RStudio. For mRNA–mRNA, mRNA–lncRNA, or lncRNA–lncRNA pairs, their *r* values were calculated to determine the significant correlation pairs.

### Identification of hub molecules with molecular complex detection

The mRNA–mRNA interactions were analyzed with Cytoscape software (version 3.2.1; National Resource for Network Biology) to obtain the network. The criteria of hub-molecule searching were set as the molecular complex detection (MCODE) score > 6 and statistical significance of *p* < 0.05.

### The ceRNA network and identification of an integrated lncRNA–RNA binding protein-mRNA signature

LncRNA–miRNA–mRNA interaction networks and lncRNA–RNA binding protein-mRNA interaction networks were generated from the large-scale CLIP-Seq data by starBase v 2.0 (http://starbase.sysu.edu.cn/mirCircRNA.php). Cytoscape 3.4.0 (http://www.cytoscape.org/) was used to visualize the network.

### Cell lines and cell culture

OC cells TOV-21G, A2780, and SKOV3, and normal cells IOSE80 were purchased from Keibai Academy of Science (Nanjing, China). RPMI-1640 medium was used to culture TOV-21G cells with 5% CO_2_ atmosphere at 37 °C. DMEM medium (Corning, NY, USA) plus 10% fetal bovine serum (FBS, Gibco) was used to culture SKOV3, A2780, and IOSE80 cells with 5% CO_2_ atmosphere at 37 °C. TOV-21G, A2780, and SKOV3 cells belong to human epithelial ovarian cancer cell line, and IOSE80 cells as normal control were also from ovarian surface epithelium. Most of TCGA ovarian cancer patients were serous cystadenocarcinoma. Among the selected ovarian cancer cell lines, SKOV3 cells were derived from the ascitic fluid from a 64-year-old Caucasian female with ovarian cancer, and were moderately well-differentiated adenocarcinoma, which was consistent with ovarian primary cells (serous cystadenocarcinoma). In addition, TOV-21G (clear-cell carcinoma) and A2780 (secretion of mucin-like substances into a culture medium) were also used to verify WGCNA results in different types of cell lines. TOV-21G, A2780, SKOV3, and IOSE80 were all derived from ovary epithelial tissue. It is reasonable to use those cell lines for validation of WGCNA results.

### RNA extraction and qRT-PCR

The ovarian cells (4 × 10^6^) were used to extract total RNA through the following steps: (i) the ovarian cells were washed with PBS (3×); (ii) a volume (1 ml) of TRizol Reagent (Invitrogen) was used to lyse cells (10 min, ice); (iii) 200 μl chloroform was added to each tube with sufficient mixing; (iv) after resting for 5 min on ice, they were centrifuged (12,000 r/min, 15 min); (v) the same volume of isopropanol was added to supernatant with sufficient mixing; (vi) after resting for 15 min on ice, they were centrifuged (12,000 r/min, 15 min); (vii) a volume (1 ml) of ethanol (*v*/*v* = 75%) was added to precipitate, and then centrifuged (12,000 r/min, 5 min); and (viii) after removing ethanol, 20 μl RNA enzyme-free water was added to dissolve RNA precipitate. Each total RNA was reversely transcribed into cDNA for quantitative real-time PCR (qRT-PCR) analysis with SYBR Premix ExTaq kit (TaKaRa). For the reverse transcription reaction system: (i) add 2 μl 5× gDNA Eraser buffer, 1 μl 5× gDNA Eraser buffer, 500 ng total RNAs, and RNase-free water up to 10 μl at 42 °C for 2 min. (ii) Add 1 μl PrimeScript RT Enzyme Mix I, 1 μl RT Primer Mix, 2 μl 5× PrimeScript buffer, 4 μl RNase-free water to reaction solution from the first step at 37 °C for 15 min, 85 °C for 5 s, and save at 4 °C. qRT-PCR reaction system contained 5 μl SYBR buffer, 4 μM primers (forward and reverse primers), 2 μl RNase-free water, and 1 μl cDNA. Beta-actin was set as an internal control for gene quantification. The numbers of technical and biological replicates were at least three times for each gene with qRT-PCR analysis. Table 1 contained those RNA molecules that were assessed on the cell lines and their corresponding primers.

**Table 1:** The list of RNA molecules that were assessed on the cell lines

RNA type	Primer name	Primer sequence (from 5′ to 3′)
Reference gene	β-actin-F	AGGGGCCGGACTCGTCATACT
	β-actin-R	GGCGGCACCACCATGTACCCT
lncRNA	ITGB2-AS1-F	AAGGCAGGTGAGTGTAGGAAGGAG
	ITGB2-AS1-R	ACCACGCAGAGGAAGGCAGAG
	OTUD6B-AS1-F	GGCAGAGATCTGAATCGTGAGGAG
	OTUD6B-AS1-R	GTAGCATGGAGGTGGCACATAGC
	PSMG3-AS1-F	TGGAACGGTGAAGGAATCTGAAGC
	PSMG3-AS1-R	GTGGCTGTGAGGTGTGGATGTG
	LINC00324-F	CTGCAACGAAGAGCTAGGTCCAAG
	LINC00324-R	GGTTACCGACTTGGTGCCATTCC
	LINC01503-F	TTCGAACGCCTCTGACAAGTGTG
	LINC01503-R	GTCCACTCCAGATGGTCCTCAGG
	HOTAIRM1-F	TGGAGTGCTGGAGCGAAGAAGAG
	HOTAIRM1-R	TCCTGGATGCGATTCGTCCTCTC
	LINC01547-F	AGGCCAAGAGACAACAGCGATTAC
	LINC01547-R	GCCAAGTGTGGACTCAGAGCTTC
	SH3PXD2A-AS1-F	CTGAAGCAGCACTGTGGAGATCC
	SH3PXD2A-AS1-R	GCTCATCTCGCTGGCAGACTG
	HCP5-F	GGTTGGTGCAGATGGTGATAGGAC
	HCP5-R	CACAGGCTTGGCACTGCTCTC
	MIR31HG-F	AGCAGGTCTCCAGGTGTTCCAG
	MIR31HG-R	GGAAGTCAGCCAGTTGCAGAAGG
	MIR155HG-F	ACCTTACCTGTCACCTTGGCTCTC
	MIR155HG-R	CAGCAAGCCTTCAGCACTCAGAG
	ZBED5-AS1-F	ACTCCGCCTCTCGAAGTGATGG
	ZBED5-AS1-R	TGACTCGCACAGATGGTGTTCATG
mRNA	LAMA1-F	GGCACACGGTCAAGACAGACTATG
	LAMA1-R	CACATCCAGCATGGTTCCATCTCC
	KIAA1549-F	CTTCACTCTCGAAGCAACAGTC
	KIAA1549-R	ACAGTTGTGATCAGATAGGCAT
	TCF7L1-F	ATCTCCAGCACACTTGTCTAAT
	TCF7L1-R	TTCCTGTCTTTGGATCGATCTC
	DNMT3A-F	GAATGTGCCAAAACTGCAAGAA
	DNMT3A-R	GTTCCAGGGGTCTTCCTTAATG
	EFS-F	CTCTGAGAGCACAGGTCAG
	EFS-R	TAGTGAGCAGGGTAGTGAATTG
	SBK1-F	TCACCAACAGCCTCTCCTCCAG
	SBK1-R	GCGCTTCACCGTGTCCTCAG
	PLCG1-F	ACCGTCATGACTTTGTTCTACT
	PLCG1-R	AATTTCACGAATGTCAATGGCC
	C10orf82-F	TGCCGAGAGCCAAGGTCACTG
	C10orf82-R	CCTCTCCGTGATCTCCAGGAAGTC
	TUBB2B-F	TGAAGGAGGTGGACGAGCAGATG
	TUBB2B-R	CCGTGCTGTTGCCGATGAAGG

*F* forward, *R* reverse

### Statistical analysis

All original data were downloaded from TCGA dataset and analyzed by R software 3.4.1 with WGCNA package (https://www.r-project.org/). For the pair of module–trait relationship and gene significance (GS) for module membership (MM) based on WGCNA analysis, Pearson correlation coefficient (*r*) was calculated. Benjamini-Hochberg for multiple testing and false discovery rate (FDR) were used to correct the *p* value. *p* value for GO enrichment analysis of mRNAs in mRNA-based yellow co-expression module was obtained by two-sided hypergeometric test and corrected by Benjamini-Hochberg. The Kaplan–Meier survival curves of hub molecules in brown and yellow co-expression modules were tested by log rank (Mantel–Cox). Correlation analyses for hub molecules in brown and yellow co-expression modules were analyzed by Pearson correlation coefficient, respectively. The identified 21 hub-mRNAs and 16 survival-associated lncRNAs were input to multivariate regression module in SPSS 20 software (*p* < 0.05). Each experiment for qRT-PCR was repeated in totality three times, and the means and standard deviations (mean ± SD) were calculated. The differences between groups for in vitro studies were analyzed by *t* test in SPSS 13.0 (SPSS Inc., Chicago, USA), with statistical significance (*p* < 0.05).

## Results

### Construction of co-expression modules of OC

**Fig. 1:**
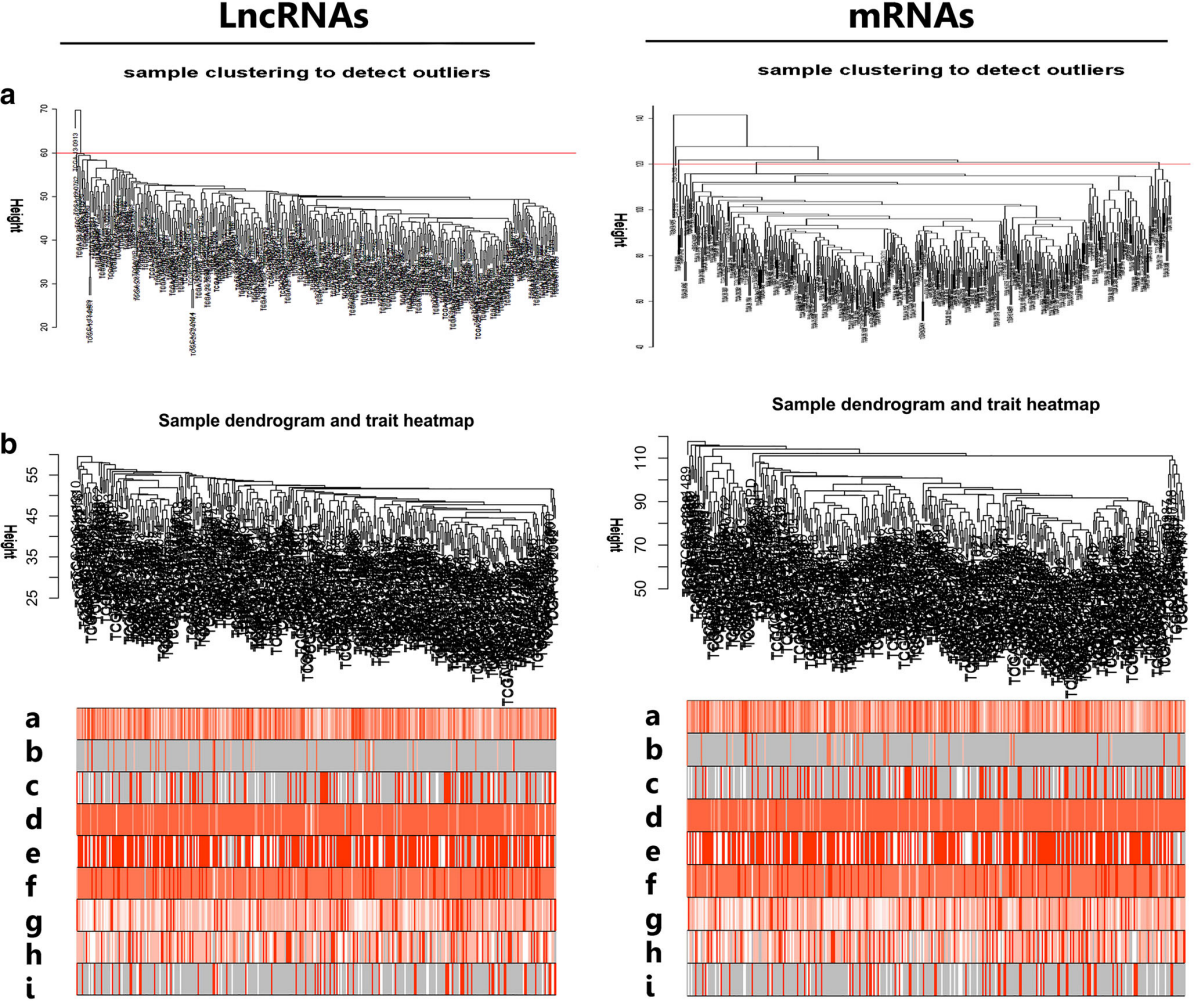
Sample cluster analysis based on lncRNA data (left column) and mRNA data (right column). **A** Sample clustering to detect outliers based on lncRNA data and mRNA data. The red line represents the cutoff of data filtering in the step of data preprocessing. **B** Sample dendrogram and trait heatmap based on lncRNA and mRNA expression data and clinical data: a, age at initial pathologic diagnosis; b, Karnofsky performance score; c, lymphatic invasion; d, histologic grade; e, cancer status; f, clinical stage; g, tissue source site; h, tumor residual disease; i, vascular invasion

**Fig. 2:**
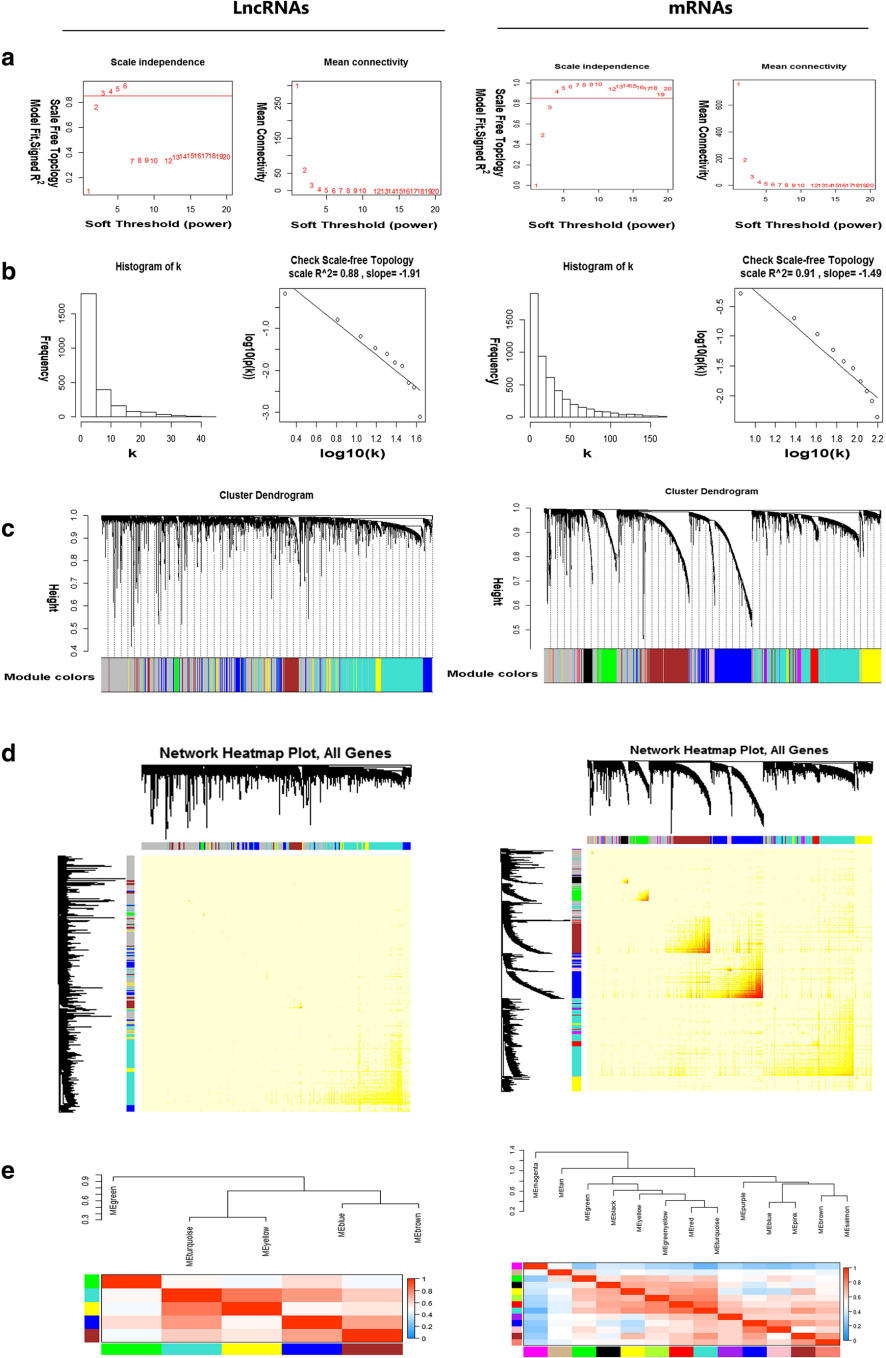
Construction of co-expression modules of ovarian cancers based on lncRNA data (left column) and mRNA data (right column). **A** Analysis of network topology for various soft-threshold powers, including the scale-free fit index (*y*-axis) and the mean connectivity (degree, *y*-axis). **B** Check scale-free topology, and here the adjacency matrix was defined using soft-thresholds with beta = 3 for lncRNA data, and with beta = 4 for mRNA data. **C** Clustering dendrograms of lncRNAs or mRNAs, with dissimilarity based on topological overlap, together with assigned module colors. As a result, six co-expression modules (co-expression green module, co-expression turquoise module, co-expression yellow module, co-expression blue module, co-expression brown module, co-expression gray module) were constructed from lnRNA data, and 14 co-expression modules from mRNA data (co-expression magenta module, co-expression tan module, co-expression green module, co-expression black module, co-expression yellow module, co-expression green-yellow module, co-expression red module, co-expression turquoise module, co-expression purple module, co-expression blue module, co-expression pink module, co-expression brown module, co-expression salmon module, co-expression gray module). **D** The heatmap depicts the topological overlap matrix (TOM) among all lncRNAs or all mRNAs. **E** Visualizing the gene network using a heatmap plot

After removing the missing value of gene expression from raw data, quantile normalization, and WGCNA package filtration, the datasets with 2562 lncRNAs and the top 5000 mRNAs were selected for WGCNA analysis. The expression values of 2562 lncRNAs in 352 OC samples (Supplementary Table 1) and 5000 mRNAs in 359 OC samples (Supplementary Table 2) were utilized to establish co-expression modules with WGCNA package. The clinical characteristics of these 370 (combined those 352 and 359 OC samples with removal of the replicates) eligible patients were summarized (Supplementary Table 3). The samples were clustered by the FlashClust tool with average linkage method and Pearson’s correlation method. Sample clustering identified outliers based on lncRNA and mRNA data, respectively. The red line was the cutoff value to filter data (Fig. 1A). All the samples were in the clusters after removing outliers in the samples based on lncRNA data and mRNA data (Fig. 1B). Sample dendrogram and trait heatmap were plotted based on lncRNA (or mRNA) expression data and mRNA clinical data (Fig. 1B). The approach of algorithm made every sample in different clusters, and showed clinical-data distribution. The power value was the most critical parameter to mainly influence the average connectivity degree and the independence of each co-expression module. Firstly, the power *β* was selected in lncRNA and mRNA groups, respectively. When *β* = 3, the scale *R*^2^ was 0.88 to obtain a higher average connectivity degree in the lncRNA group. When *β* = 4, the scale *R*^2^ was 0.91 to obtain a higher average connectivity degree in the mRNA group (Fig. 2A and B). Thereby, the *β* determined distinct gene co-expression modules in OCs. The cluster dendrogram of all selected genes was clustered with the adjacency matrix. These co-expression modules were shown (Fig. 2C). These co-expression modules were distributed within a range from small to large due to the number of included genes. Their interactions were analyzed between co-expression modules.

Heatmap was plotted to reflect topological overlap. Each column and row represented a gene. Low topological overlap was shown in light color, and higher topological overlap was shown in progressively darker red. Each module was shown in darker squares. The network heatmap plot of all genes and module assignment were shown (Fig. 2D). Hierarchical clustering revealed module eigengenes to summarize the modules. Dendrogram branches were grouped together with positively correlated eigengenes. One color module eigengene was shown in each column and row of heatmap: low adjacency was negative correlation in blue, and high adjacency was positive correlation in red. The red squares along the diagonal were defined as meta-modules (Fig. 2E).

### Gene co-expression modules corresponding to clinical traits

**Fig. 3:**
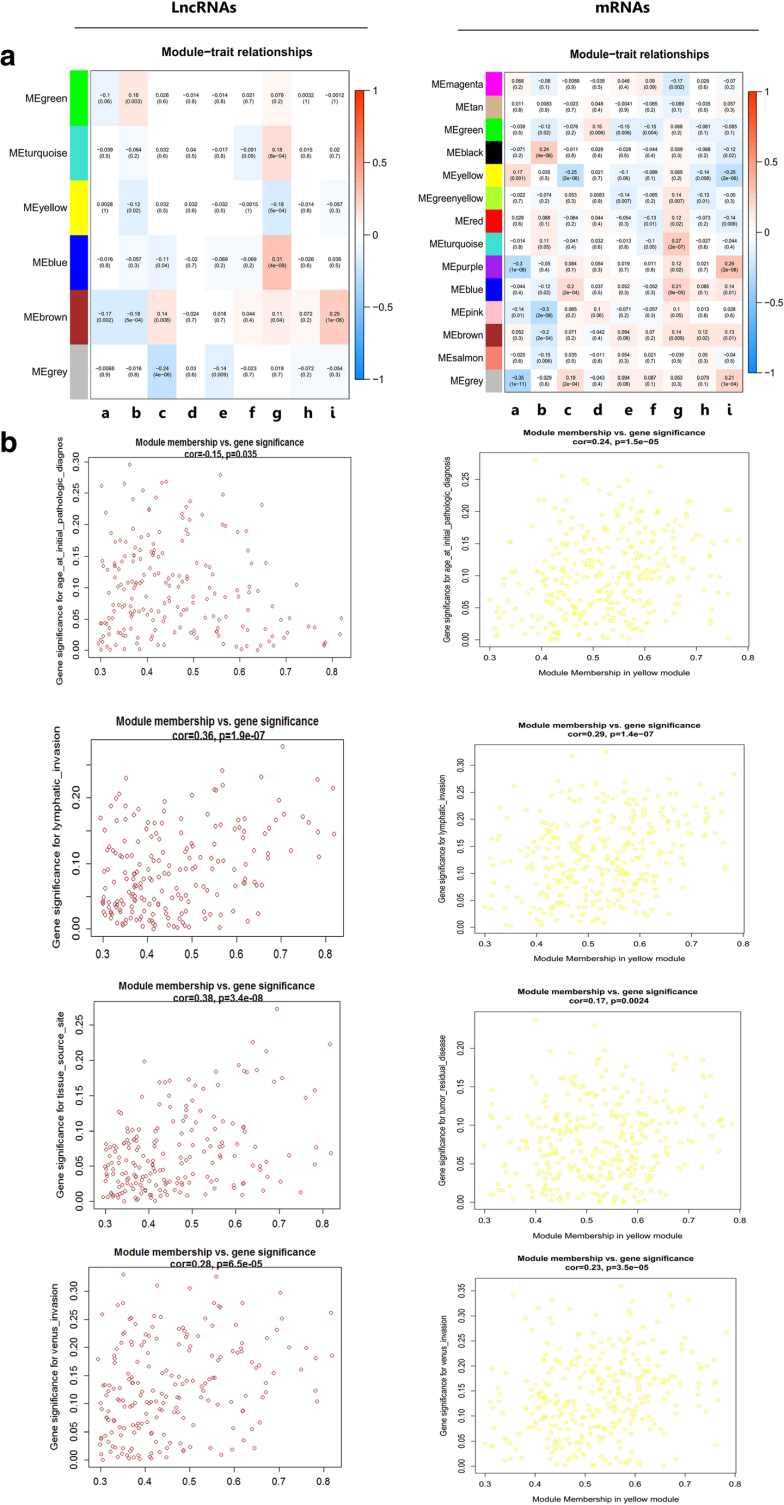
Analysis of module-trait relationships of ovarian cancer based on lncRNA data (left column) and mRNA data (right column). **A** Module-trait associations. Each row corresponds to a module eigengene, and column to a trait. a, age at initial pathologic diagnosis; b, Karnofsky performance score; c, lymphatic invasion; d, histologic grade; e, cancer status; f, clinical stage; g, tissue source site; h, tumor residual disease; i, vascular invasion. **B** The scatterplot of gene significance (GS) vs. module membership (MM) in the lncRNA-based brown co-expression module, or in the mRNA-based yellow co-expression module

The association analysis was performed between common expression eigengene pattern in co-expression module and the particular clinical trait dataset from the TCGA database, including age at initial pathologic diagnosis, Karnofsky performance score, lymphatic invasion, histologic grade, cancer status, clinical stage, tissue source site, tumor residual disease, and vascular invasion (Fig. 3A). Heatmap was constructed for the correlation between clinical traits and module eigengenes in ovarian cancer, with *r* and *p* values. Based on heatmap of module-trait relationship for lncRNA, gene co-expression module and clinical traits demonstrated that the green module in Fig. 3A was significantly associated with OC Karnofsky performance score, which indicated the close relation of lncRNAs in this co-expression module to the activities of daily life (independent, semi-independent, or dependent) after the patients received treatment. The blue and turquoise modules in Fig. 3A were significantly associated with OC tissue source site, which indicated heterogeneity of gene expression; namely, gene expression was different in different tissues, different even in the same organ tissue. Based on the heatmap of module–trait relationship for mRNA, gene co-expression module and clinical traits demonstrated that the black module in Fig. 3A was significantly associated with OC Karnofsky performance score, which indicated the close relation of mRNAs in this co-expression module to the activities of daily life (independent, semi-independent, or dependent) after the patients received treatment. The blue, green, and purple modules in Fig. 3A were significantly associated with OC lymphatic invasion, histologic grade, and vascular invasion, respectively, which indicated that mRNAs in those co-expression modules were closely related to OC metastasis. Various co-expression modules were related to the clinical trait of tissue source site in module–trait relationship for mRNA, including green, yellow, red, turquoise, purple, and brown modules (Fig. 3A), which indicated that a large heterogeneity exists from different origins of ovary cancer sites. The brown module in the lncRNA group and the yellow module in the mRNA group in Fig. 3A were chosen as key modules for further study according to correlation coefficient (*r*) and *p* values, and those two co-expression modules were associated with multiple clinical traits. For lncRNAs, the correlation analysis of gene co-expression module and clinical traits demonstrated that the brown modules that contained 168 RNAs (Fig. 3A; Supplementary Table 4) were significantly associated with OC clinical traits, including age at initial pathologic diagnosis (*r =* − 0.17, *p* = 2.0E− 03), Karnofsky performance score (*r* = − 0.18, *p* = 5E− 04), clinical stage (*r* = 0.14, *p* = 8.0E− 08), tissue source site (*r* = 0.11, *p* = 4.0E− 02), and vascular invasion (*r* = 0.25, *p* = 1.0E− 06). For mRNAs, the correlation analysis between clinical traits and gene co-expression modules demonstrated that the yellow modules that contained 318 mRNAs (Fig. 3A; Supplementary Table 5) were significantly associated with OC clinical traits, including age at initial pathologic diagnosis (*r* = 0.17, *p* = 1.0E− 03), lymphatic invasion (*r* = −0.25, *p* = 2E− 06), tumor residual disease (*r* = − 0.14, *p* = 8.0E− 03), and vascular invasion (*r* = − 0.25, *p* = 2E− 06). Furthermore, the scatterplot was plotted between GS and MM in lncRNA-based brown module and mRNA-based yellow module, respectively. Scatterplot was constructed between MM in *x*-axis and GS in *y*-axis for lncRNA-based brown module, and mRNA-based yellow module. In the module–trait relationships, the higher MM value means the higher GS, which suggested hub genes in brown co-expression module or yellow co-expression module were also highly associated with selected clinical characteristics. The results revealed that MM in lncRNA-based brown module was significantly correlated with age at initial pathologic diagnosis (*r* = − 0.15, *p* = 3.5E− 02), lymphatic invasion (*r* = 0.36, *p* = 1.9E− 07), tissue source site (*r* = 0.38, *p* = 3.4E− 08), and vascular invasion (*r* = 0.28, *p* = 6.5E− 05) (Fig. 3B), and that MM in mRNA-based yellow module was significantly correlated with age at initial pathologic diagnosis (*r* = 0.24, *p* = 1.5E− 05), lymphatic invasion (*r* = 0.29, *p* = 1.4E− 07), tumor residual disease (*r* = 0.17, *p* = 2.4E− 03), and vascular invasion (*r* = 0.23, *p* = 3.5E− 05) (Fig. 3B).

### Functional enrichment analysis of mRNAs in an mRNA-based co-expression module

KEGG pathway analysis revealed ten statistically significant signaling pathways to involve mRNAs identified in mRNA-based yellow co-expression module (Supplementary Table 6); and interestingly, OC cells had the enhanced dependence on multiple signaling pathways, including Hippo signaling pathway, basal cell carcinoma, melanogenesis, Wnt signaling pathway, pathways in cancer, proteoglycans in cancer, aldosterone synthesis and secretion, gap junction, ovarian steroidogenesis, and signaling pathways regulating pluripotency of stem cells. For example, organ growth depends on a series of cell biological processes, including cell proliferation, cell division, and programmed cell death. Hippo signaling pathway inhibits cell proliferation and induces apoptosis [28], which is becoming increasingly important in the study of uncontrolled cell division in cancer. The notably enriched mRNAs in Hippo signaling pathway included WNT5A, DLG4, LEF1, TEAD2, PARD6G, FZD2, BMP7, WNT6, TCF7L1, FZD7, and BMP6. The Wnt signaling pathway plays important roles in many diseases. CTNNB1 mRNA profile alteration, which encodes β-catenin protein, was found in melanoma, breast colorectal, lung, prostate, and other cancers. One study found that Wnt ligand proteins (Wnt 1, Wnt2, and Wnt7A) were significantly upregulated in esophageal cancer, glioblastoma, and OC [29]. Other changed proteins included SFRP4, ROR1, ROR2, WIF1 Wnt5A, and TCF/LEF family. The notably enriched mRNAs in Wnt signaling pathway included WNT5A, GPC4, PLCB4, LEF1, FZD2, BAMBI, WNT6, TCF7L1, and FZD7. Hormone hypothesis in OCs recognized that hormones were OC risk factors, including androgens, gonadotropin, insulin-like growth factor I, progesterone, estrogens, and insulin, and androgens were associated with increased risk of ovarian-origin cancers [30]. The notably enriched mRNAs in ovarian steroidogenesis pathway included CYP17A1, CYP11A1, STAR, and BMP6.

**Fig. 4:**
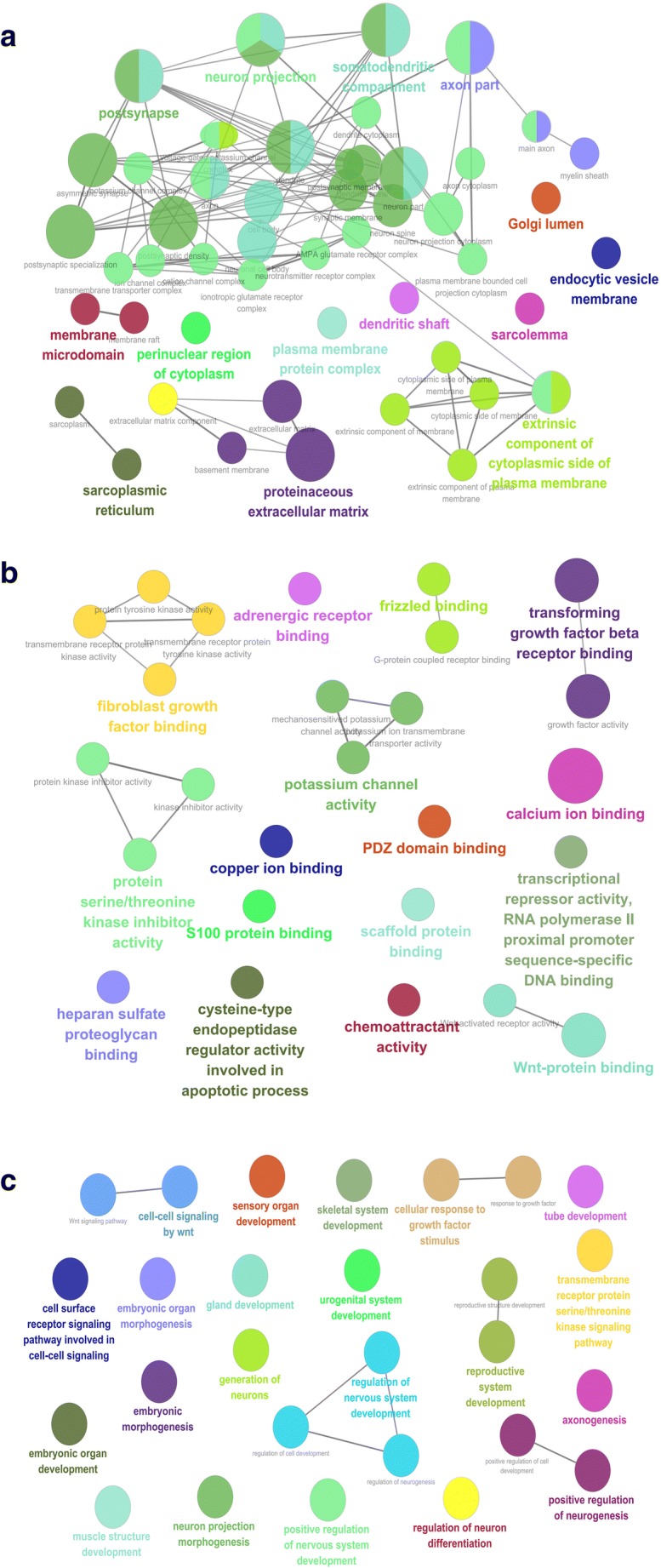
GO analysis involved in mRNAs in mRNA-based yellow co-expression module. **A** Cellular component derived from mRNAs in the mRNA yellow co-expression module. **B** Molecular function derived from mRNAs in the mRNA yellow co-expression module. **C** Biological process derived from mRNAs in the mRNA yellow co-expression module

GO enrichment analysis of mRNAs in mRNA-based yellow co-expression module revealed cellular component (CC) (Fig. 4A; Supplementary Table 7), molecular function (MF) (Fig. 4B; Supplementary Table 8), and biological process (BP) (Fig. 4C; Supplementary Table 9). For CC enrichment, the mRNAs in mRNA-based yellow co-expression module were mainly distributed in postsynapse, neuron projection, somatodendritic compartment, axon part, Golgi lumen, endocytic vesicle membrane, dendritic shaft, plasma membrane protein complex, membrane microdomain, perinuclear region of cytoplasm, sarcoplasmic reticulum, and proteinaceous extracellular matrix. For MF enrichment, the mRNAs in mRNA-based yellow co-expression module were mainly distributed in Wnt-protein binding, adrenergic receptor binding, frizzled binding, transforming growth factor beta receptor binding, fibroblast growth factor binding, potassium channel activity, calcium-ion binding, PDZ-domain binding, copper-ion binding, S100 protein binding, protein serine/threonine kinase inhibitor activity, scaffold protein binding, chemoattractant activity, heparan sulfate proteoglycan binding, and cysteine-type endopeptidase regulator activity involved in apoptotic process. For BP enrichment, the mRNAs in mRNA-based yellow co-expression module were classified into ten groups to involve major BPs, including urogenital system development, mesoderm formation, mesenchyme development, cardiac muscle tissue development, endocrine system development, kidney morphogenesis, embryonic organ development, epithelial tube morphogenesis, morphogenesis of a branching epithelium, gland morphogenesis, and neuroepithelial cell differentiation.

### Hub genes and survival-associated genes

The intramodular connectivity was to sum connection strengths with other module genes, and was divided by the maximum intramodular connectivity. High intramodular connectivity was defined as MCODE score > 6 and *p* < 0.05, whose genes were looked as intramodular hub genes. A total of 21 hub-mRNAs were identified from 318 mRNAs in mRNA-based yellow co-expression module, including FBN3, EFS, MSI1, TCF7L1, FXYD6, ZNF423, SULT1C4, SBK1, TRO, SMO, SALL2, TUBB2B, PLCG1, LRP4, KIAA1549, PHC1, RHOBTB1, DNMT3A, TMEFF1, LAMA1, and C10orf82.

**Fig. 5:**
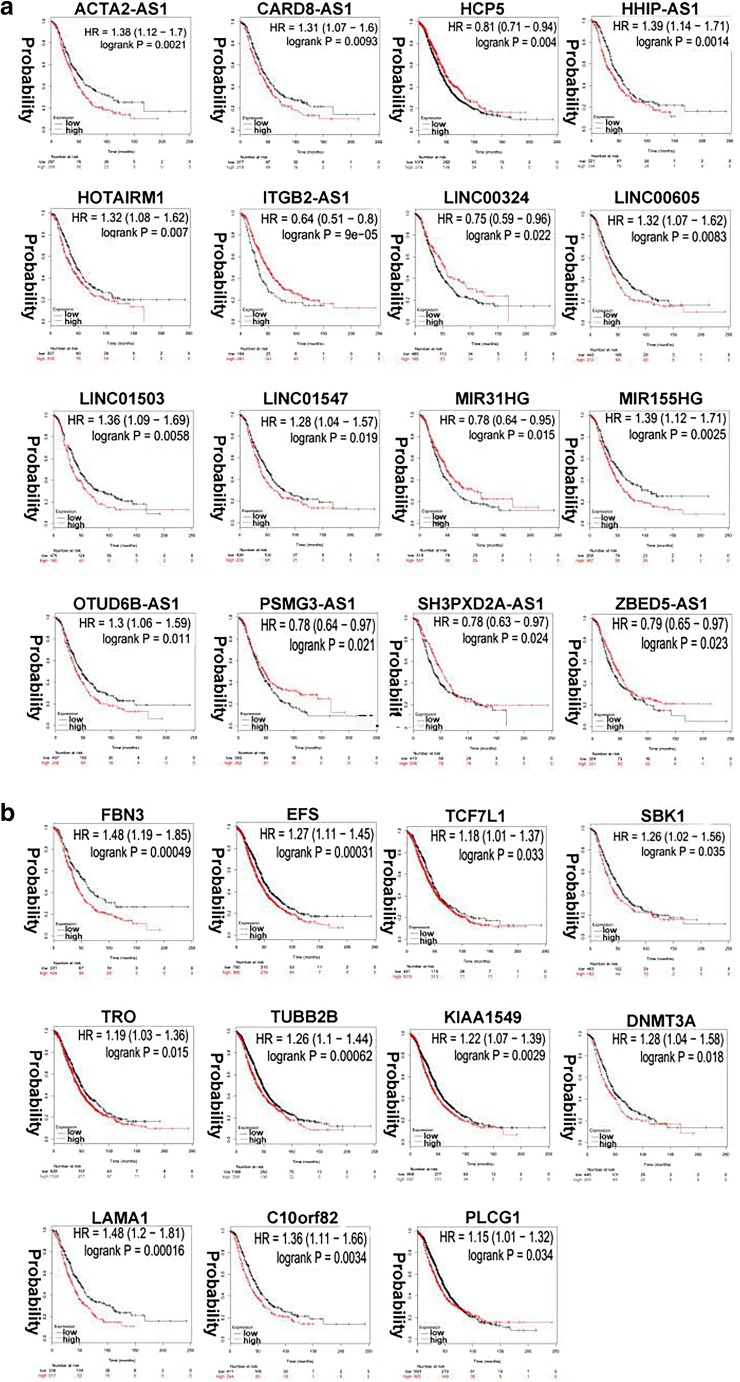
Analysis of overall survival–related lncRNAs (**a**) and mRNAs (**b**) in ovarian cancers

The K-M plot analysis revealed that 11 out of 21 hub-mRNAs in the mRNA-based yellow co-expression module were significantly related to OC overall survival (*p* < 0.05), including FBN3 (HR = 1.48, *p* = 4.9E− 04), EFS (HR = 1.27, *p* = 3.1E− 04), TCF7L1 (HR = 1.18, *p* = 3.3E− 02), SBK1 (HR = 1.26, *p* = 3.5E− 02), TRO (HR = 1.19, *p* = 1.5E− 02), TUBB2B (HR = 1.26, *p* = 6.2E− 04), PLCG1 (HR = 1.15, *p* = 3.4E− 02), KIAA1549 (HR = 1.22, *p* = 2.9E− 03), DNMT3A (HR = 1.33, *p* = 7.0E− 03), LAMA1 (HR = 1.48, *p* = 1.6E− 04), and C10orf82 (HR = 1.36, *p* = 3.4E− 03) (Fig. 5). The K-M plot analysis revealed that 16 out of 168 lncRNAs in lncRNA-based brown co-expression module were significantly related to OC overall survival (*p* < 0.05), including ACTA2-AS1 (HR = 1.38, *p* = 2.1E− 03), CARD8-AS1 (HR = 1.31, *p* = 9.3E− 03), HCP5 (HR = 0.81, *p* = 4.0E− 03), HHIP-AS1 (HR = 1.39, *p* = 1.4E− 03), HOTAIRM1 (HR = 1.33, *p* = 7.0E− 03), ITGB2-AS1 (HR = 0.64, *p* = 9.0E− 05), LINC00324 (HR = 0.75, *p* = 2.2E− 02), LINC00605 (HR = 1.32, *p* = 8.3E− 03), LINC01503 (HR = 1.36, *p* = 5.8E− 03), LINC01547 (HR = 1.28, *p* = 1.9E− 03), MIR31HG (HR = 1.39, *p* = 2.5E− 03), MIR155HG (HR = 0.78, *p* = 1.5E− 02), OTUD6B-AS1 (HR = 1.3, *p* = 1.1E− 02), PSMG3-AS1 (HR = 0.78, *p* = 2.1E− 02), SH3PXD2A-AS1 (HR = 0.78, *p* = 2.4E− 02), and ZBED5-AS1 (HR = 0.79, *p* = 2.3E− 02) (Fig. 5).

**Fig. 6:**
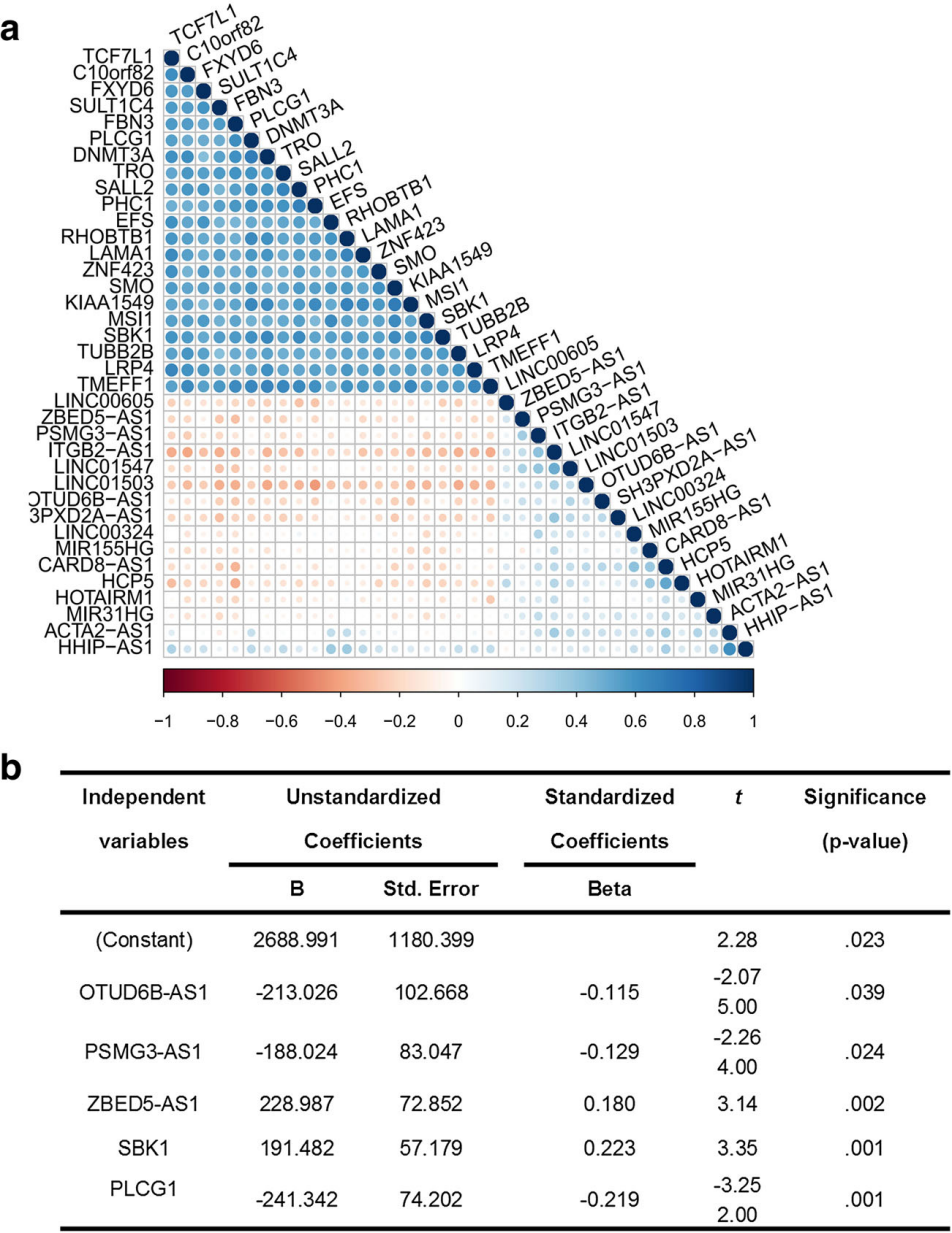
Establishment of co-expression models and survival-related regression model based on 16 survival-related lncRNAs and 21 hub-mRNAs in ovarian cancers. **A** Co-expressions between 16 lncRNAs and 21 hub-mRNAs. **B** Survival-related regression model based on 16 lncRNAs and 21 mRNA hub molecules as independent variables and overall survival (OS: days) as dependent variable (ANOVA, df = 5, *F* = 5.936, *p* = 0.000)

Moreover, RStudio software was used to determine co-expressions of lncRNAs and mRNAs (Fig. 6A), and obtain their correlation coefficients (Supplementary Table 10) and *p* values (Supplementary Table 11). Some highly correlated (|correlation coefficient| ≥ 0.4, *p* < 0.05) mRNA–lncRNA, mRNA–mRNA, or lncRNA–lncRNA pairs were identified, including EFS and HHIP-AS1, HHIP-AS1 and TCF7L1, RHOBTB1 and HHIP-AS1, ACTA2-AS1 and HHIP-AS1, CARD8-AS1 and HCP5, LINC00324 and CARD8-AS1, ITGB2-AS1 and LINC01547, LRP4 and TCF7L1, SALL2 and TRO, DNMT3A and PLCG1, and SMO and KIAA1549. Those high-correlation hub-mRNAs and hub-lncRNAs are worthy for further studying to demonstrate their encoded spatiotemporal dynamics.

In addition, survival risk score system was constructed with 21 identified hub-mRNAs and 16 survival-associated lncRNAs using the multivariate regression module in SPSS 20 software. A statistically significant regression equation (Fig. 6B; *p* < 0.05) was generated to calculate the survival risk score: survival risk score = (− 0.115 × expression level of OTUD6B-AS1) + (− 0.129 × expression level of PSMG3-AS1) + (0.18 × expression level of ZBED5-AS1) + (0.223 × expression level of SBK1) + (− 0.219 × expression level of PLCG1). For this survival risk score system, a higher score indicated a longer survival time or a lower mortality risk for OC patients.

### Network analysis and RT-qPCR confirmed the identified molecules

**Fig. 7:**
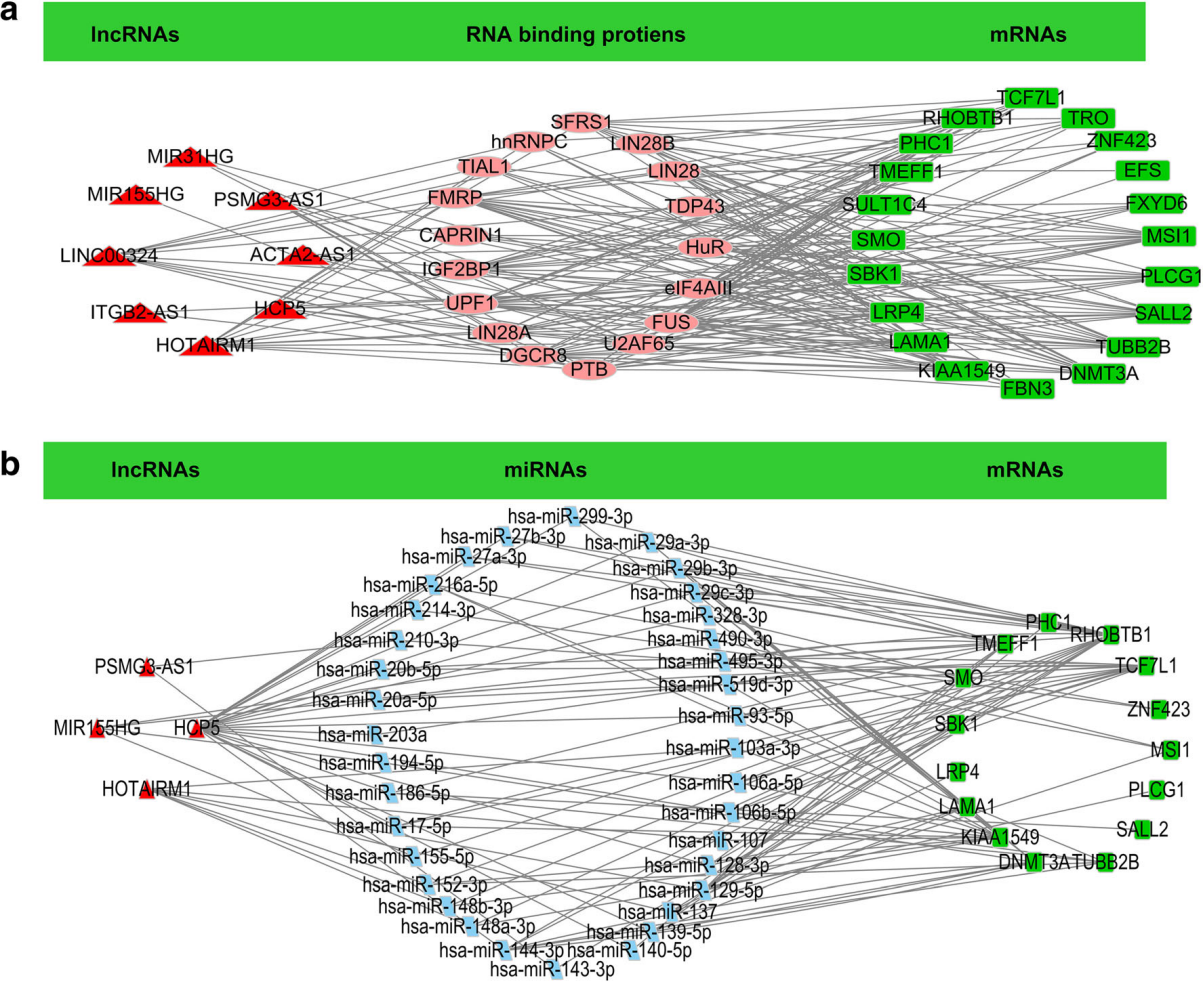
Constructions of lncRNA–RNA binding protein-mRNA network (**A**) and lncRNA–miRNA–mRNA network (**B**) based on 16 survival-related lncRNAs and 21 hub-mRNAs

lncRNA–RNA binding protein-mRNA network analyses were used to determine whether lncRNAs regulate hub-mRNAs through RNA-binding proteins. This type of network analysis found that 8 lncRNAs (ACTA2-AS1, HCP5, HOTAIRM1, ITGB2-AS1, LINC00324, MIR155HG, MIR31HG, and PSMG3-AS1), 17 RNA-binding proteins (HuR, eIF4AIII, FUS, U2AF65, PTB, FMRP, LIN28A, UPF1, IGF2BP1, DGCR8, CAPRIN1, SFRS1, TIAL1, hnRNPC, LIN28B, LIN28, and TDP43), and 20 hub-mRNAs (MSI1, PLCG1, SALL2, TUBB2B, DNMT3A, FBN3, KIAA1549, LAMA1, LRP4, SBK1, SMO, SULT1C4, TMEFF1, PHC1, RHOBTB1, TCF7L1, TRO, ZNF423, EFS, and FXYD6) were involved in the network (Fig. 7A). A ceRNA network analysis was used to determine whether lncRNAs regulate hub-mRNAs through miRNAs. Moreover, the ceRNA network analysis found that 4 lncRNAs (HOTAIRM1, HCP5, PSMG3-AS1, and MIR155HG), 35 miRNAs (miR-106a-5p, miR-106b-5p, miR-128-3p, miR-139-5p, miR-140-5p, miR-144-3p, miR-17-5p, miR-186-5p, miR-203a, miR-20a-5p, miR-20b-5p, miR-214-3p, miR-216a-5p, miR-27a-3p, miR-27b-3p, miR-299-3p, miR-29a-3p, miR-29b-3p miR-29c-3p, miR-328-3p, miR-519d-3p, miR-93-5p, miR-103a-3p, miR-107, miR-129-5p, miR-137, miR-148a-3p, miR-148b-3p, miR-152-3p, miR-155-5p, miR-194-5p, miR-490-3p, miR-495-3p, miR-143-3p, miR-210-3p), and 15 hub-mRNAs (KIAA1549, TCF7L1, TUBB2B, LAMA1, RHOBTB1, TMEFF1, PHC1, PLCG1, SBK1, LRP4, MSI1, DNMT3A, SALL2, SMO, and ZNF423) were involved in a ceRNA network (Fig. 7B).

**Fig. 8:**
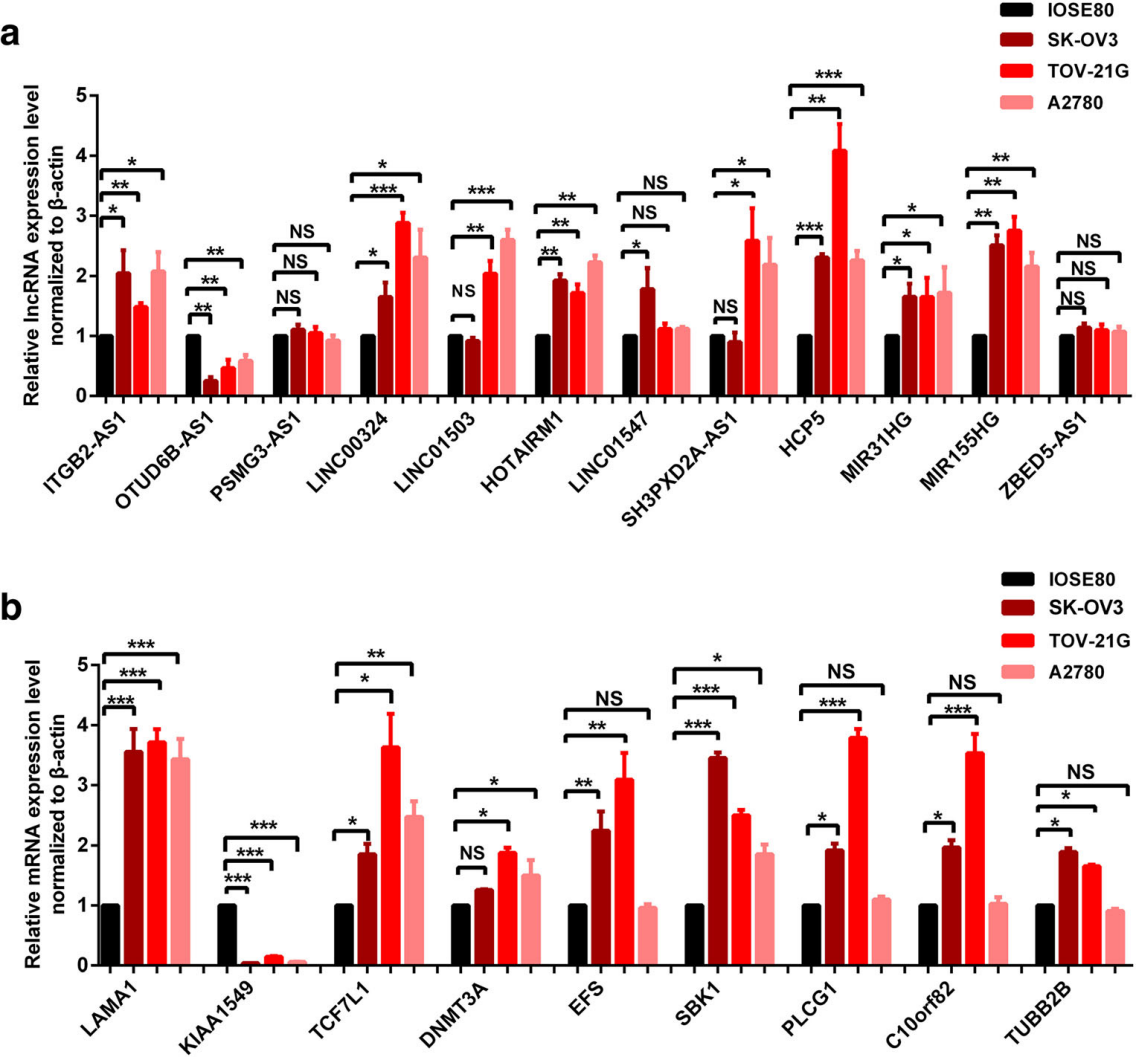
qRT-PCR analysis of 16 survival-related lncRNAs (**A**) and 11 survival-related hub-mRNAs (**B**) in OC cell models compared with control cells. *p < 0.05; **p < 0.01; ***p < 0.001. *n* = 3

Furthermore, qRT-PCR was used to validate the expressions of OC survival-associated lncRNAs and hub-mRNAs that are from WGCNA analysis, including 16 lncRNAs (ITGB2-AS1, OTUD6B-AS1, PSMG3-AS1, LINC00324, LINC01503, HOTAIRM1, LINC01547, SH3PXD2A-AS1, HCP5, MIR31HG, MIR155HG, ZBED5-AS1, LINC00605, ACT2-AS, CARD8-AS1, and HHIP-AS1) and 11 hub-mRNAs (LAMA1, KIAA1549, TCF7L1, DNMT3A, EFS, SBK1, PLCG1, C10orf82, TUBB2B, TRO, and FBN3) in 3 cultured OC cells and 1 control cell (Fig. 8). Among them, the too low expressions of four lncRNAs (LINC00605, ACT2-AS, CARD8-AS1, and HHIP-AS1) cause their difficulty to be quantified with qRT-PCR. The results showed that no significant difference was found for three lncRNAs (PSMG3-AS1, LINC01547, and ZBED5-AS1) between OC cells (SK-OV3, TOV-21G, and A2780) and control cell IOSE80 (*p* > 0.05), whereas significant difference was found for nine survival-associated lncRNAs (ITGB2-AS1, OTUD6B-AS1, LINC00324, LINC01503, HOTAIRM1, SH3PXD2A-AS1, HCP5, MIR31HG, and MIR155HG) (Fig. 8A), and nine survival-associated hub-mRNAs (LAMA1, KIAA1549, TCF7L1, DNMT3A, EFS, SBK1, PLCG1, C10orf82, and TUBB2B) (Fig. 8B) between OC cells and control cells.

## Discussion

OC is a high-mortality gynecologic malignant tumor [31]. Although significant progress has been made in OC diagnosis, the 5-year overall survival rate for OC patients is still very poor due to recurrence and metastasis [32]. Its effective early-stage diagnosis biomarkers and therapeutic targets remain poor. It is necessary to identify novel diagnostic markers or therapeutic targets for understating the complex molecular mechanisms and effective management of OCs. Molecular pattern recognition is an effective strategy for unpaired diagnosis and treatment of OCs [33], which promoted the shift of traditional medical concept from a single-parameter model to a multi-parameter systematical model [11]. Compared to high-degree complex and dynamic proteoforms in a proteome [34], RNAs in a transcriptome are much simpler and relatively stable, and also RNAs include mRNAs, lncRNAs, and miRNAs. Therefore, investigation of RNA biomarkers has important scientific merits for effective OC management. This study for the first time identified malignant clinical phenotype (clinical traits)-associated lncRNAs and mRNAs using lncRNA and mRNA data from the TCGA database on OCs. These findings provide novel insights into lncRNA-related networks in OCs and useful resource for identification of biomarkers in OCs.

Six co-expression modules were identified with 2562 lncRNAs (Supplementary Table 1), and 14 co-expression modules were identified with 5000 mRNAs (Supplementary Table 2), from 370 human OC samples with the WGCNA method, and co-expression modules were applied to investigate the associations between transcriptomes and clinical traits in OCs. WGCNA showed various advantages compared with other bioinformatics methods because it focused on correlations between clinical traits and co-expression modules, whose results had much higher biological significance and reliability [35]. Genes that were clustered in the same module were considered to be associated with each other in biological function. Therefore, identification of biologically related modules and hub genes to serve as biomarkers for diagnosis or treatment is very possible. Further analysis found that 168 lncRNAs in lncRNA-based brown co-expression module (Supplementary Table 4) were significantly associated with OC clinical traits, including age at initial pathologic diagnosis, Karnofsky performance score, clinical stage, tissue source site, and vascular invasion; and that 318 mRNAs in mRNA-based yellow co-expression module (Supplementary Table 5) were significantly associated with OC clinical traits, including age at initial pathologic diagnosis, lymphatic invasion, tumor residual disease, and vascular invasion. Moreover, lncRNA–RNA binding protein-mRNA network and lncRNA–miRNA–mRNA network were the interaction patterns, which provided the molecular explanation of OC patients. Recently, the sponge roles of lncRNAs and lncRNA–miRNA–mRNA network have been widely accepted [36]. For instance, lncRNA FOXD2-AS1 controlled the miR-485-5p/KLK7 axis to enhance papillary thyroid cancer progression, which revealed that FOXD2-AS1 acted as a ceRNA to increase the expression of KLK7 through sponging miR-485-5p in papillary thyroid cancer [37].

The CooLGeN database (http://ci.smu.edu.cn/CooLGeN/ Home.php) was analyzed with key word “cancer” to help understand our findings in OCs compared with other cancers. The results in OCs were consistent with other cancer studies, and new findings were made; for example, five lncRNAs (MIR31HG34, HOTAIRM1, MIR155HG, ITGB2-AS1, HCP5, and SH3PXD2A-AS134) were reported in other different cancers [38], which confirmed the reliability of our newfound biomarkers. A more interesting thing was that our found mRNA biomarkers for OC by the WGCNA method, including DNMT3A, SMO, SALL2, TRO, FBN3, MSI1, and SBK1, were also reported in other OC studies [39]. It demonstrated that WGCNA was a reliable tool to identify OC biomarkers. Moreover, some of our identified lncRNAs and mRNAs, including ACTA2-AS1, CARD8-AS1, HHIP-AS1, LINC00324, LINC00605, LINC01503, LINC01547, OTUD6B-AS1, PSMG3-AS1, ZBED5-AS1, EFS, TCF7L1, FXYD6, ZNF423, SULT1C4, TUBB2B, PLCG1, LRP4, KIAA1549 PHC1, RHOBTB1, TMEFF1, LAMA1, and C10orf82, have never been reported in previous OC biomarker studies, which is worthy of further investigation for discovery of novel biomarkers for OCs. Most of the traditional studies only focused on single-one factor or single-one gene in cancers. However, the reality is that cancer is involved in multiple molecular events [8]. This study avoided the single-one parameter model, and recognized the multi-molecule pattern biomarker to improve the specificity and accuracy in prediction, diagnosis, prognosis, and therapy for OC patients.

### Strengths and limitations

WGCNA is an effective approach to detect intrinsic links between prognostic factors and functional gene clusters. The identified OC-specific lncRNAs and mRNAs were selected to construct multi-molecule biomarkers in ovarian cancers. However, one might also note that, first, there are partial ovarian cancer patients with incomplete clinical information (Supplemental Table 3), which might affect the clinical assessment of the research result. Second, the identified lncRNAs and mRNAs were confirmed in cell models; it might be also necessary to be further validated in the large-scale clinical samples for their real application in the ovarian cancers.

## Conclusions and expert recommendations

WGCNA was an effective method to identify cancer-related lncRNAs and mRNAs in the publicly free-access TCGA database for predictive, preventive, and personalized medicine (PPPM) in OCs. A set of lncRNAs and mRNAs were identified to associate with malignant phenotypes, and theoretically induced OC malignant phenotypes through regulating cancer-related signal pathways. Further, lncRNA–miRNA–mRNA networks and lncRNA–RNA binding protein-mRNA networks were identified based on those identified lncRNAs and mRNAs in OCs to clarify the molecular mechanisms of lncRNAs regulating mRNAs. It is the first comprehensive study to investigate lncRNA–miRNA–mRNA networks and lncRNA–RNA binding protein-mRNA networks in OCs based on the TCGA database, and some important lncRNAs and mRNAs were confirmed in OC cell models. These findings are an important source to develop new biomarkers and anti-cancer targets for early-stage diagnosis, effective therapy, and prognostic assessment to achieve effective and reliable personalized treatment of OC patients.

We recommend strengthening the understanding and application of transcriptome (lnRNAs, miRNAs, and mRNAs) in OC research and clinical practice for PPPM in future OC care. Here, one must realize that PPPM is the future direction for OC care [14, 15]. OC is a chronic and complex disease associated with multiple causes, multiple processes, and multiple consequences, which is involved in multiple levels of molecular alterations in genome, transcriptome, proteome, metabolome, and radiome [8, 9]. Multiomics has driven the rapid development of PPPM in OCs. Multiomics-based pattern biomarker is the effective and affordable approach to reveal the real molecular mechanism and discover therapeutic targets and diagnostic and prognostic markers for effective treatment of OCs [10, 11]. This study focused on the transcriptome-based pattern biomarkers in OCs, which has opened the window to further insight into the molecular world changed in OCs, important roles of non-coding RNAs including lncRNAs that played a role in regulation of transcription and translation of genes to affect alterations in proteome, metabolome, and even other biological processes, and further promotes one to study OCs in the comprehensive level of multiomics in the future, especially integrative analysis of transcriptomics with proteomics and metabolomics [16], for improving services to OC patients in the PPPM context.

## Electronic supplementary material

ESM 1 (XLSX 10662 kb)

ESM 2 (XLSX 21160 kb)

ESM 3 (XLSX 331 kb)

ESM 4 (XLSX 31 kb)

ESM 5 (XLSX 40 kb)

ESM 6 (XLSX 27 kb)

ESM 7 (XLSX 31 kb)

ESM 8 (XLSX 39 kb)

ESM 9 (XLSX 79 kb)

ESM 10 (XLSX 42 kb)

ESM 11 (XLSX 43 kb)
